# Fractionation and Distribution of Rare Earth Elements in Marine Sediment and Bioavailability in *Avicennia marina* in Central Red Sea Mangrove Ecosystems

**DOI:** 10.3390/plants10061233

**Published:** 2021-06-17

**Authors:** Abdullahi Bala Alhassan, Mohammed Othman Aljahdali

**Affiliations:** 1 Department of Biological Sciences, Faculty of Science, King Abdulaziz University (KAU), P.O. Box 80203, Jeddah 21589, Saudi Arabia; 2 Department of Biology, Faculty of Life Sciences, Ahmadu Bello University, Zaria 810001, Nigeria

**Keywords:** rare earth elements, fractionation, bioaccumulation, sediment, mangrove, *Avicennia marina*

## Abstract

Rare earth element fractionation and distribution in the coastal ecosystem have been of significant concern and are recognized worldwide as emerging micro-pollutants. However, unlike other metals such as trace elements, little is known about their uptake by aquatic plants such as the mangrove *Avicennia marina,* especially in the central Red Sea. We investigated the fractionation of rare earth elements in six mangrove ecosystems in the central Red Sea and bioavailability in mangrove *A*. *marina*. The concentrations of rare earth elements, sediment grain sizes, multi-elemental ratios, geo-accumulation index (I_geo_) and bioconcentration factor (BCF) vary significantly (*p* < 0.05) across the six mangrove ecosystems. Higher concentrations of rare earth elements were recorded at Al Lith (LT) (101.53 mg/kg) and South Jeddah (SJ) (73.38 mg/kg) mangrove ecosystems. However, multi-elemental ratio R_(M/L)_ reveals positive values. In contrast, multi-elemental ratio R_(H/M)_ reveals negative values corresponding to fractionation patterns enriched with medium rare earth elements and heavy rare earth elements depletion across the six mangrove ecosystems. BCF values for rare earth elements were <1, but Lutetium (0.32) had the highest BCF among the rare earth elements, suggesting an efficient accumulation of Lutetium than any other rare earth elements. The scale of I_geo_ revealed strong contamination (4 ≤ I_geo_ ≥ 5) of sediment with Lanthanum, Cerium, Praseodynium, Samarium, Godolinium, Holmium, Erbium, Ytterbium, and moderate contamination with Thulium, Terbium, and Dysprosium (1 ≤ I_geo_ ≤ 3). Principal component analysis showed that clay silt sediment grain size influences rare earth element concentrations in the central Red Sea. Our results provide new evidence for rare earth element fractionation and accumulation in sediment and the potential use of mangrove *A. marina* for rare earth element monitoring in mangrove ecosystems in the central Red Sea.

## 1. Introduction

The rare earth elements (REE) are made up of 17 elements comprising 15 lanthanides, beginning from Lanthanum, which is the lightest, and to heaviest Lutetium (Lanthanum, Cerium, Praseodymium, Neodymium, Promethium, Samarium, Europium, Godolinium, Terbium, Dysprosium, Holmium, Erbium, Thulium, Ytterbium, and Lutetium) [1,2]. Except for Ce and Eu that can exist as Ce (IV) and Eu (II), REEs are usually trivalent elements. Even though Yttrium lacks 4f electrons, its ionic radii and geochemical behavior are similar to that of Ho, and Yttrium is usually considered an REE and defined together with Scandium as heavy REE [2]. REE are usually divided into two groups, namely light rare earth elements (LREE), which comprises of La to Eu (La, Ce, Pr, Nd, Sm, Eu), and heavy rare earth elements (HREE), which include Gd to Lu (Gd, Tb, Dy, Ho, Er, Tm, Yb, Lu) and Yttrium [1]. They have similar geochemical behaviors and provide an understanding of complex processes of a geochemical nature that single proxies cannot readily discriminate due to their coherent and predictable characteristics [3,4]. The geochemical evolution of chemical weathering in marine ecosystems is mostly evaluated by exploring the fractionation of REEs [5,6].

Rare earth elements are recognized globally as emerging micro-pollutants in aquatic ecosystems [7,8]. This is due to the fact that modern technologies involving different applications of high technology used REE because of their exclusive physicochemical properties [9]. Therefore, the distribution of REE in most coastal ecosystems such as mangroves is expected not to be natural as they have already been influenced by anthropogenic activities [2,6,8]. The low REE solubility and possibility of predicting their occurrence and their unchanged behavior have led to better information on their sources and distribution in an environment [10]. The abundance and distribution of REE are used as tracers of geochemical processes and anthropogenic contamination in natural aquatic ecosystems, making them essential in scientific research [11,12].

Mangrove ecosystems are very important and provide various ecological services; among the services are regulation and exchange of materials between the terrestrial and aquatic ecosystems [13]. The accumulation of materials in mangrove sediment is favored by mangrove root structure, giving the sediment a characteristic form of ability to act as sinks and sources of metals [2,14]. However, reports on bioaccumulation, the pattern of fractionation, and distribution of REE in mangrove plants such as *Avicennia marina* are few [6]. In contrast, REE bioaccumulation and their effects as expressed through physiological responses such as growth and yield quality have been established in several non-mangrove species on soils with natural concentrations of REE [15,16]. Even though Brito et al. [17] reported concentrations of REEs in salt marsh halophyte, to the best of our knowledge, investigation on REE bioaccumulation and quantitative assessments of the complete set of REE in sediment and mangrove *A. marina* are either few or lacking.

Here we report the distribution, fractionation of REEs in sediment, and bioaccumulation in mangrove *A. marina* in the central Red Sea for the first time. We sampled sediments and mangrove leaves from six mangrove ecosystems, namely Al Lith (LT), South Jeddah (SJ), Dhaban (DB), Thuwal (TH), Rabigh (RB), and Mastorah (MA) in the central Red Sea selected based on the different sources of anthropogenic activities. REE (La, Ce, Pr, Nd, Sm, Eu, Gd, Tb, Dy, Ho, Er, Tm, Yb, and Lu) were measured in sediments and leaves, and sediment grain sizes, bio-concentration factor (BCF) and geo-accumulation index (I_geo_) were determined. I_geo_ is used to measure the extent of metal contamination in sediment using the scale with seven enrichment classes [18], while BCF is used to determine the efficiency or capacity of metal uptake by plants. This way, the plant can be classified as either hypo-accumulator or hyper-accumulator. Our goal in this research is to investigate REE fractionation, contamination, and bioavailability in mangroves. Our research will serve as new evidence for REE dynamics in the central Red Sea. The values of REE in our study can also be used as background values for further investigation and designing environmental monitoring studies of these important ecosystems.

## 2. Results

### 2.1. REE Composition in Sediment and Grain Sizes

The results for sediment grain size analysis revealed significant (ANOVA, Tukey’s HSD; *p* < 0.05) variation in coarse sandy (0.063~2 mm), clay and silt (<0.063 mm), and gravels (>2 mm) contents across the six mangrove ecosystems investigated (Table 1). The minimum average content of coarse sandy grain type was at Rabigh (RB) (45.50%) mangrove ecosystem, while maximum average content was recorded at South Jeddah (SJ) (88.83%) mangrove ecosystem. Clay silt particles exhibit a contrasting pattern; their lowest value (10.53%) occurs at SJ and their highest value (54.29%) at RB, while gravel grain size ranged from 0.20% at RB and Dahban (DB) mangrove ecosystems to 0.63% at SJ (Table 1).

The average total REE content in sediment varies significantly across the mangroves (Table 2). In addition, the mean concentrations of the 14 REE (La, Ce, Pr, Nd, Sm, Eu, Gd, Tb, Dy, Ho, Er, Tm, Yb, and Lu) (mg/kg) in sediments differed substantially in the six mangroves investigated in this study. The highest concentrations of REE were recorded at Al Lith (LT) and South Jeddah (SJ), and the concentrations in LT were even higher than those at SJ. The lowest concentrations of REE were recorded in sediments from Thuwal (TH) mangrove ecosystem. The ∑REE (101.53 mg/kg) was highest at LT and lowest at TH (13.85 mg/kg) mangrove ecosystem which was about 1/7 the ∑REE at LT. However, the lighter rare earth elements (LREE and MREE) dominate the REE composition forming the major contribution to ∑REE with 9.18~82.38%, greater than the contribution by heavy rare earth elements (HREE: 8.45~13.28%). The total of LREE (La, Ce, Pr, Nd) was about 7 folds MREE (Sm, Eu, Gd) and HREE (Tb, Dy, Ho, Er, Tm, Yb, Lu) composition (Table 2).

**Figure 1:**
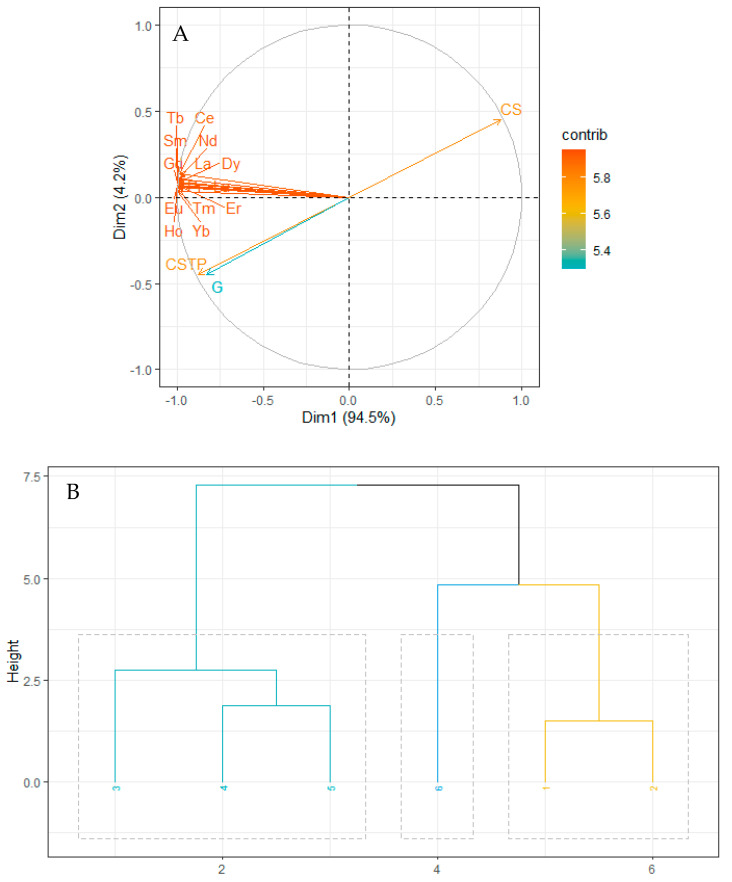
Principal Component Analysis for the relationship between REEs concentration and sediment grain sizes. (**A**) contribution to dimension and total variation and (**B**) hierarchical cluster formation. CS-Coarse sandy, CSTP-Clay and Silt particles, G-Gravels, 1-Al Lith, 2-South Jeddah, 3-Dahban, 4-Thuwal, 5-Rabigh, 6-Mastorah.

The influence of sediment grain size on REEs concentration was revealed by principal component analysis (Figure 1A,B). The total variation contributed by components 1 and 2 with the highest sources of variation was 98.7%, with components 1 and 2 accounting for 94.5 and 4.2% of the total variation, respectively. REEs, clay silt, and coarse grain particles had a high contribution (>5.6) (Figure 1A) to the total variation. However, the clay silt grain size type forms a strong positive correlation (r = 0.8771) with REEs (Figure 1A,B). Based on the relationship between REEs and grain size types, clusters were formed between the mangroves investigated; the clusters are between LT and SJ, DB, TH, and RB, while MA forms no cluster with any study site (Figure 1B).

### 2.2. REE Fractionation and Geo-Accumulation Index (I_geo_)

**Figure 2:**
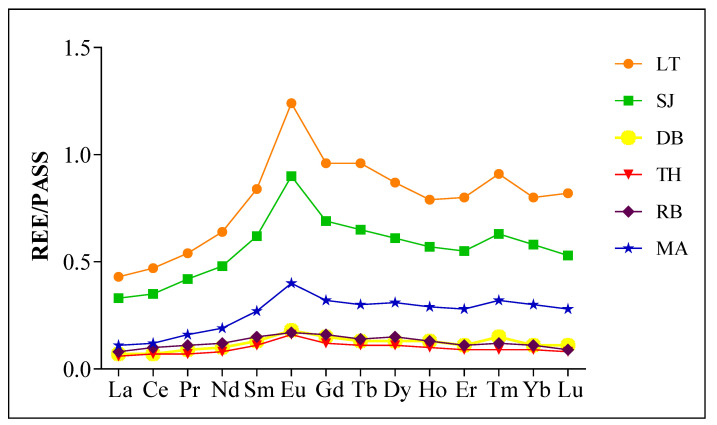
Rare earth elements/Post-Archean Australian Shale (REE/PAAS) patterns obtained for surface sediment of six mangrove ecosystems in central Red Sea.

To achieve a better understanding of REE accumulation patterns in the sediment of six mangrove ecosystems investigated, Post-Archean Australian Shale (PASS) [19] normalized REE patterns of the sediments across the mangroves were plotted (Figure 2). Relative enrichment in ∑REE and similar fractionation trends of REE were revealed by the results; (La/Yb)n range from 0.38 at MA to 0.72 at RB, with an average value of 0.57 for the six mangroves.

**Figure 3:**
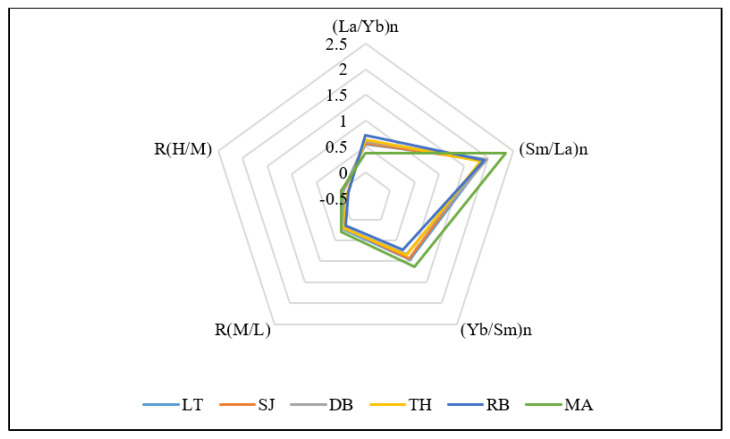
Radar chart showing the median proportion of REE fractions for six mangrove ecosystems.

For (Sm/La)n, the fractions range from 1.85 at TH to 2.34 at MA, the average value of fractions for the six mangroves was 1.98, reflecting the remarkable accumulation of LREE and MREE, and the highest median proportion among the fractions across the mangroves (Figure 3; Table 2). The lowest (0.73) (Yb/Sm)n was recorded at RB, while the highest fraction (1.12) was at MA, reflecting relatively flat HREE patterns (Figure 2). However, the average fraction (Yb/Sm)n for the mangrove ecosystems investigated was 0.91 (Table 2).

The results of multi-elemental ratios, R_(M/L)_ and R_(H/M)_ revealed higher positive values corresponding to fractionation patterns enriched with MREE and HREE depletion, as the values for R_(M/L)_ were positive and the range values for R_(H/M)_ were negative (Table 2). The lowest value (0.16) for R_(M/L)_ was recorded at RB, while the highest value (0.30) was at MA, indicating more enrichment of MREEs at MA. However, the average for the six mangroves was 0.22. R_(H/M)_ ranges from −0.15 at RB to −0.01 at MA, with an average value of −0.07 indicating HREEs depletion in the mangrove ecosystems with even more depletion at RB. Significant variation (ANOVA, Tukey’s HSD; *p* < 0.05) existed for both multi-elemental ratios (R_(M/L)_ and R_(H/M)_) across the mangrove ecosystems.

The Ce and Eu anomalies calculated during the usual expected shale-normalized concentrations of REE in order to quantify the possible existence of anomalous concentrations relative to its neighboring REE revealed ranges of Ce and Eu anomalies across the mangroves. Ce showed a small negative anomaly from the average value as the average value was close to one (0.97). However, considering the mangroves individually, anomalies of Ce at TH (1.03) and RB (1.05) were positive anomalies, while a slightly lower negative anomaly (0.90) was established at MA. For Eu, a small positive anomaly (1.31) was recorded, with slightly lower (1.09) and higher (1.38) values at RB and LT (Table 2). 

**Figure 4:**
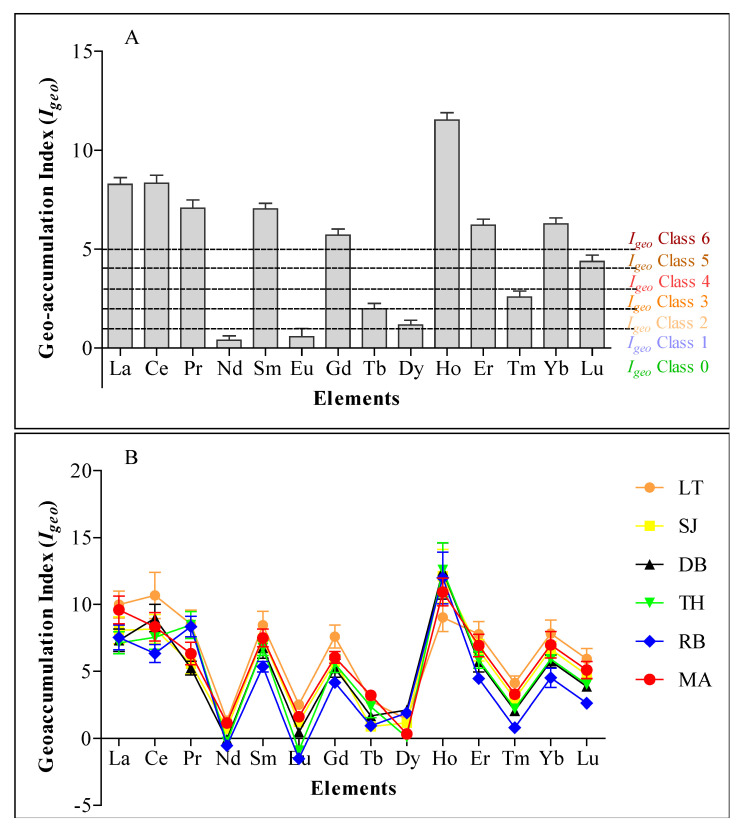
Geo-accumulation index (I_geo_) for an element (**A**) and sum total for elements at each mangrove ecosystem (**B**).

Geo-accumulation index (I_geo_) as an index of sediment quality using the seven classes of enrichment [18] revealed a strong to extreme contamination (4 ≤ I_geo_ ≥ 5) of La, Ce, Pr, Sm, Gd, Ho, Er, Yb, and Lu in all six mangrove ecosystems. The sediment was also moderately to strongly contaminated (1 ≤ I_geo_ ≤ 3) with Tm, Tb, and Dy. However, the sediments were either uncontaminated or moderately contaminated (0 ≤ I_geo_ ≤ 1) with Nd and Eu (Figure 4A,B). I_geo_ values for Nd at DB, TH, and RB, and Eu at TH and RB were negative (Figure 4B), signifying the uncontaminated status of the mangroves with Nd and Eu.

### 2.3. REE in Mangrove Avicennia marina and Bio-Concentration Factor (BCF)

**Figure 5:**
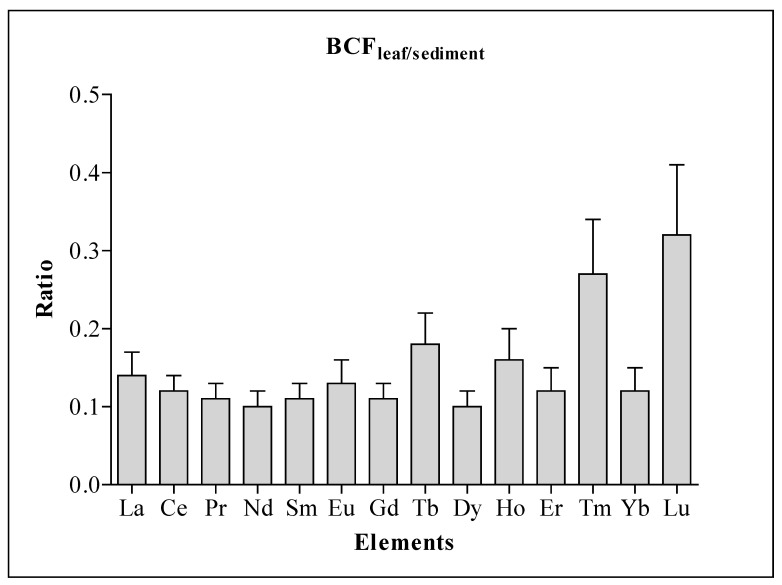
Bioconcentration factor (BCF) for REE concentrations in six mangrove ecosystems.

Significant variation (ANOVA, Tukey’s HSD; *p* < 0.05) in REE concentrations in *A. marina* leaves was established across the mangrove ecosystems investigated except for Eu, Tb, Ho, Er, Tm, Yb, and Lu (Table 3). The pattern of distribution of REE in *A. marina* leaves across the mangrove ecosystems was similar to that of sediment, with higher concentrations of REEs at LT and SJ, and lower concentrations at TH. The highest ∑REE in *A. marina* leaves at LT (3.56 mg/kg) was about 1/29 that in sediment, while the lowest at TH (2.57 mg/kg) was about 1/5 of the concentration in sediment (Table 2 and Table 3). This suggests that the ∑REE in sediment at LT was about 29 folds concentration in mangrove leaves, and for TH, it was 5 folds concentration in mangrove leaves. However, Lu recorded the highest (0.32) BCF value, followed by Tm (0.27), while Nd and Dy recorded the lowest (0.10). All the BCF for the REEs were less than 1 (Figure 5).

## 3. Discussion

### 3.1. Influence of Sediment Grain Size on REE Concentrations and Fractionation

The REE concentrations and sediment grain size types, together with the results from data analysis, reveal that the sediment grain size is an important factor that determines the accumulation of REEs in sediments from the six mangrove ecosystems investigated. The influence of grain size on concentrations of elements and their distribution has been reported to partly reflect the impacts of the hydrodynamic environment, as hydrodynamics causes the transportation, re-suspension, and deposition of sediment of allochthonous sources into aquatic ecosystems such as mangrove ecosystems [20,21]. However, a large surface area as a property of fine grain particles can increase the sorption of elements [22]. This develops an understanding of the strong positive correlation between clay silt sediment particles and REEs concentrations. In addition, higher contribution to the total variation by clay silt and coarse sediment grain sizes, and REEs (La, Ce, Pr, Nd, Sm, Eu, Gd, Tb, Dy, Ho, Er, Tm, Yb, Lu) as revealed by the contribution plot could also be due to the abovementioned reasons [21,23]. Apart from environmental hydrodynamics, other geomorphological factors such as regional siltation, sediment, and sediment grain size impact REEs distribution [24,25]. This gives more insight into the influence of grain size-types on the distribution and concentrations of REEs in the studied mangrove ecosystems [26]. Previous studies have reported variations in metal concentrations and the influence of sediment grain size on their distribution [21,26,27,28]. Fine-grain mangrove sediment has also been observed elsewhere to have the capacity to adsorb REE [29].

Higher concentrations of REEs in sediments of Al Lith (LT) and South Jeddah (SJ) mangrove ecosystems could be associated with industrial activities in these regions of the kingdom [6]. However, it is important to note that ∑REE in LT was about 7 folds the value recorded in Thuwal (TH) mangrove sediments with little or no anthropogenic activities. Elsewhere, in the coastal areas along the Egyptian coast of the Red Sea, lower ∑REE (47.55 mg/kg), which is about 1/2 of ∑REE in LT (101.53 mg/kg) and SJ (75.38 mg/kg) mangrove ecosystems in our study was reported. In addition, the ∑REE at TH (13.85 mg/kg) with the lowest value among the six ecosystems investigated in our study was about 1/3 of that at the Egyptian coast of the Red Sea [30].

Similar observations on depletion of HREE relative to lighter rare earth elements were reported in previous studies [2,17]. LREE and MREE are likely more reactive than HREE [31]. They are better associated with solid phases because they are more pronounced complexation with ligands on surfaces of the colloids and the particles. However, HREE depletion in the sediment is attributed to their higher tendency to form stable soluble carbonates and organic complexes with ligands than LREE and MREE [32]. These phenomena could cause the removal and/or preferential of LREE [2].

### 3.2. Fractionation of REE and Geo-Accumulation Index (I_geo_)

The Post Archean Australian Shale (PAAS) [19] is utilized widely to normalize REE concentrations in marine sediments to reveal the fractionation of REEs comparatively to the source and ease comparison between findings. However, the information generated from REE fractionation is used as tracers to determine whether the impacted mangrove sediment and the sources of contamination influence the chemistry of the environment and the biodiversity [8].

Even though REE enrichment based on ∑REE was deduced earlier, the need to determine precisely LREE, MREE, and HREE enrichment in mangrove ecosystems considered in this study is important. Besides, REE fractionation base on (La/Yb)n, (Sm/La)n, and (Yb/Sm)n has been used and La, Sm, and Yb were chosen as representatives of LREE, MREE, and HREE, respectively [6,33]. Notable higher values of PAAS normalized (Sm/La)n (1.98) than (Yb/Sm)n indicate the predominance of lighter REEs (LREE and MREE) over HREE in the mangrove ecosystems. In addition, higher values of (La/Yb)n (0.72) in Rabigh (RB), and (Sm/La)n (2.34) and (Yb/Sm)n (1.12) in MA indicates the predominance of LREE at RB, and MREE and HREE at Mastorah (MA). In terms of fractionation, a ratio equal to 1 indicates no fractionation, while a ratio <1 and >1 signifies depletion and enrichment, respectively [6,34,35]. This was supported by positive and negative values of multi-elemental ratios R_(M/L)_ and R_(H/M),_ signifying fractionation patterns enriched in lighter REEs (MREE and LREE), and depletion of HREE [11]. In corroboration with our results, REE fractionation was reported in the Pichavaram mangrove ecosystem, giving reference to higher absorption of lighter REEs into clay-silt sediment of the mangroves [22]. This supports the strong positive correlation between clay silt sediment grain size (r = 0.8771) and lighter REE (La, Ce, Pr, Nd, Sm, Eu, and Gd) in this study (Figure 1A). However, it is essential to note that sediment deposition in high sedimentation regime and rapid burial may decrease the time frame of exposure to dissolved REE with sediment. This could lead to restriction in the adsorptive capacity of the sediment and possible depletion of REE, causing variations in REE concentrations in sediment [2].

A sediment quality index such as I_geo_ is a good indicator of metal contamination level and the influence of anthropogenic activities on the accumulation of metals in sediment [13,25,36]. The strong to extreme contamination (4 ≤ I_geo_ ≥ 5) of some REE using I_geo_ values recorded in the mangroves investigated in this study may be owed to anthropogenic sources and their products in these mangroves such as industrialization and agricultural waste containing pesticide and fertilizers [9,14,37,38]. It is important to note that REEs are widely used as fertilizer and are sometimes applied directly on a large scale on crops for growth, yield, and improve quality; however, they may increase REE concentration and contamination of soil [9]. In addition, the utilization of REE in modern industries involve in different technological applications in the production of various materials and finished products has been on the increase, leading to contamination of the immediate natural ecosystems due to improper disposal of industrial effluents [9]. Moderate contamination (1 ≤ I_geo_ ≤ 3) of Tm, Tb, and Dy suggests that these REEs originated mainly from anthropogenic activities and crustal material. At the same time, the negative values of I_geo_ for Nd at DB, TH, and RB, and Eu at TH and RB indicate they are from local natural sources [39]. Elsewhere in China, high contamination of REE was attributed to anthropogenic sources such as industries involved in iron and steel smelting [40].

### 3.3. REE in Mangrove Avicennia marina and Bio-Concentration Factor (BCF)

Bioconcentration factor (BCF) is commonly used to determine metal accumulation in plants due to plant-sediment interaction [13]. However, the BCFs < 1 for the REEs in this study indicate hypo-accumulation by mangrove *A. marina* or the presence of effective detoxification or exclusion mechanism in *A. marina* [6,41]. In corroboration with our results, BCFs for REEs were reported to be less than 1 in mangroves at pristine islands of Indian Sundarban; however, the highest BCF (0.32) recorded in our study was about three times the highest BCF (0.10) recorded in Indian Sundarban [6]. Differences in BCF values could be due to the concentration of bioavailable forms of REE in sediment, which can substantially influence the phytoextraction of REE [38,42]. The amount of bioavailable REE could also form the primary reason for the significant variation in REE concentration in mangrove leaves across the six mangrove ecosystems, even though the mangroves are of the same species. Several studies have also revealed that REE phytoextraction increases with an increase in sediment concentration of REE, and differences in the environment, either due to anthropogenic activities or chemical weathering, might influence sequestration of REE [43,44,45].

## 4. Materials and Methods

### 4.1. Study Area

The Red Sea comprises an area of mangroves approximately 135 Km^2^ and the mangroves are distributed to the northern boundary at 28.207302° N [46]. The central Red Sea is associated with an arid environment with high temperatures and sparse rainfall. In the central Red Sea, Saudi Arabia, some mangrove habitats appear as a narrow fringe supporting halophytes. Some of these mangroves could sometimes be flooded, especially those located along the shore and adjacent to sand flats [47,48].

**Figure 6:**
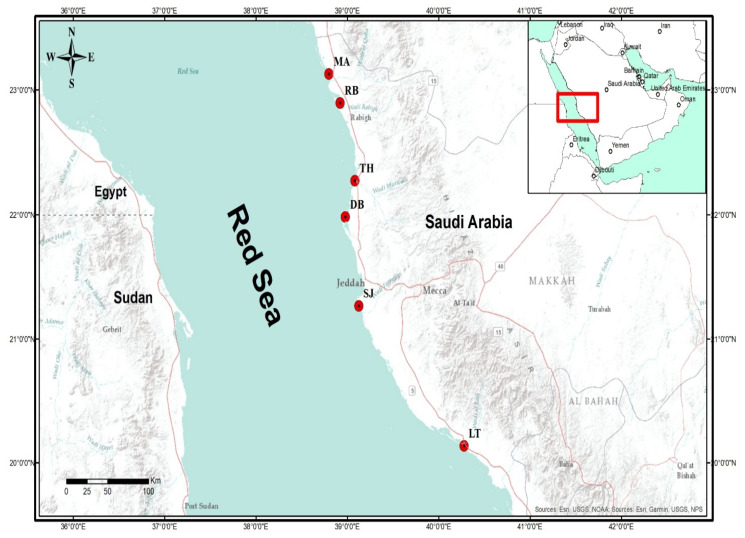
Map showing six mangrove ecosystems in central Red Sea, Saudi Arabia. Al Lith (LT); South Jeddah (SJ); Dahban (DB); Thuwal (TH); Rabigh (RB); Mastorah (MA).

The distribution and abundance of mangroves and anthropogenic activities were used to select the mangrove ecosystems (Figure 6) sampled. Six (6) mangrove ecosystems in Al lith (LT), South Jeddah (SJ), Dahban (DB), Thuwal (TH), Rabigh (RB), and Mastorah (MA) were selected to achieve our objectives. Monospecific stands of the *Avicennia marina* dominated these mangrove ecosystems. Some of the activities noticed inside and close to the mangroves are: 

LT (20°08′~18.70′′ N, 40°16′~41.74′′ E): Production and extraction of living resources involving a huge aquaculture industry and fishing, capital dredging, receive land runoff containing agricultural waste. 

SJ (20°15’~43.92′′ N, 40°25′~11.37′′ E): High population with over 3 million inhabitants, fishing, and location of the main seaport, established power plant, tourism, and recreation activities, dredging for maintenance and receive land runoff containing agricultural waste. 

DB (21°59′~05.1′′ N, 38°58′~42.9′′ E): Fishing activities in the mangroves and recreation activities in the mangrove catchment. 

Thuwal Island (TH) (22°16′~36.99′′ N, 39°05′~00.34′′ E): Desalination plant close to the mangroves, tourism and recreation activities, and fishing. 

Rabigh lagoon (RB) (22°53′~51.86′′ N, 38°55′~13.25′′ E): Large petrochemical complex (refinery) is located at the catchment, receive land runoff containing agricultural waste, decreased water circulation, and livestock activities such as camel grazing. 

Mastorah (MA) (23°07′~46.49′′ N, 38°47′~56.99′′ E): Absence of anthropogenic sources of chemical pollution in the mangroves, but ~50 km to the north is an industrial activity.

### 4.2. Sampling and REE Analysis

A total of 180 samples (30 samples in each mangrove ecosystem) were collected from six mangrove ecosystems in the central Red Sea, Saudi Arabia. Surface sediments (top 0–20 cm) and mature leaves of mangrove *A. marina* were sampled twice monthly, from May 2019 to April 2020, from LT, SJ, DB, TH, RB, and MA mangrove ecosystems. At the time of sample collection, the water depth varies from 1 to 11 m. For each ecosystem, leave samples from 15 mangrove trees and 15 sediment samples were collected in replicate. Sediment samples were collected using Van Veen grab-250 cm^2^ and placed in zip lock bags inside an icebox before conveying them to the laboratory for further analyses. 

The leave samples were cleaned using deionized water, and together with sediment samples were dried in an oven at 40–45 °C for 48 h before being crushed into powder form with agate mortar and pestle and sieved through 53 μm nylon mesh. The Leaf sample was acid digested in HNO_3_ and H_2_O_2_ (3:1) at 180 °C for 45 min using 0.2 g of sieved samples. For sediment samples, 0.4 g was weighed into a 50 mL digestion vessel, and 8 mL HNO_3_ and HCl (1:1) were added. Samples were digested in Anton-Paar PE Multiwave 3000 microwave oven at 200 °C for about 1 h after the digestion vessel was kept inside the microwave [49]. The digested sample was kept in a volumetric flask at room temperature, and Ultrapure Millipore Q water was added to top up the solution to 50 mL, shaken, and allowed to settle overnight. The solution was filtered using a GF/F filter (Whatman), and the filtrate was analyzed for 14 rare earth elements (La, Ce, Pr, Nd, Sm, Eu, Gd, Tb, Dy, Ho, Er, Tm, Yb, Lu) concentrations using an Agilent 7700x dual pump Inductively Coupled Plasma-Mass Spectrometer (ICP-MS). The construction of calibration curves was achieved by analyzing standard mixture solutions comprising 14 elements at concentrations of 0.5, 1, 5, 10, 20, 50, and 100 µg/L, with 0.999 linear fitting rates. The quality control of the analytical method was assessed using standard reference materials GSS-1 and GSV-2 for sediments and leaves, respectively. To confirm sensitivity and repeatability, the solutions of known concentration used as standard solutions were placed into the sequence of samples for every eight samples. The recoveries of REEs in percentage from the accuracy of the analytical method ranged from 94.13~116.67% (Table S1, Supplementary Materials). The acceptance of analytical precision and accuracy was based only on when the standard deviation was <5% for the rare earth elements by the outcomes of replica measurements of samples and standard materials.

### 4.3. Grain Size Analysis in Sediment

The total dry weight of oven-dried sediment samples was determined. Distilled water was used for the disintegration of solidified aggregates by soaking the dried sediments for 24 h. The sediments were washed and separated into fractions of gravel (>2 mm), coarse grain (0.063~2 mm), and mud (clay and silt, <0.063 mm) by passing the sediment through 0.063 mm and 2 mm sieves. The percentages of sediment grain sizes were computed relative to the total weight after the fractions of the residue were dried at 40 °C and weighed [22,48,50].

### 4.4. Geo-Accumulation (I_geo_) and Bio-Concentration Factor (BCF)

The geo-accumulation index was measured to determine the level of REE contamination in the sediment of six mangroves investigated base on seven enrichment classes [18] (Table S2, Supplementary Materials) using the following formula:

(1) $$I_{geo}=log_{2\;}\left[\frac{C_{n}}{1.5\times B_{n}}\right]$$

where *C_n_* is the concentration of a particular REE in the sediment and *B_n_* is the geochemical background level of that REE. 1.5 is a correction factor [51] to reduce the effect of variations due to sediment’s lithology.

To determine bioaccumulation of REE in mangrove *A. marina*, bio-concentration factor (BCF) was calculated to reveal the capability of the plant to accumulate REE using the formula:

(2) $$BCF=\frac{C_{leaves}}{C_{sediment}}$$

where *C_leaf_* and *C_sediment_* are the concentrations of a given REE in leaves and sediment.

### 4.5. Data Analyses

One-way analysis of variance (ANOVA) was used to compare mean REE concentrations in sediments and leaves, BCF, I_geo_, and sediment grain sizes across the six mangrove ecosystems. Tukey’s HSD post-hoc test was used to separate mean concentrations when statistical significance (*p* < 0.05) was recorded. Before the analysis of variance, homogeneity of variance and a test of normality were carried out using Levene’s homogeneity of variance and Shapiro-Wilk tests. Principal component analysis (PCA) was used to determine the relationship between sediment grain sizes and REE concentrations in sediments. R for Windows (v. 4.0.3) was used for the analyses.

The concentrations of REE were normalized (n) to the Post Archean Australian Shale (PAAS) [19] and the pattern and fractionation of REE were characterized using the ratios such as La/Yb, Sm/La, and Yb/Sm. Multi-elemental ratios were computed as proposed by Duvert et al. [11] and Noack et al. [52] using the formulas:

(3) R(ML)=logMREEnLREEn=log[(Gdn+Tbn+Dyn)3(Lan+Prn+Ndn)3]

(4) R(HM)=logHREEnMREEn=log[(Tmn+Ybn+Lun)3(Gdn+Tbn+Dyn)3]

where *R_(M/L)_* is the ratio between medium and light REE, *R_(H/M)_* is the ratio between heavy and medium REE, and n implies PAAS-normalized concentrations.

Ce and Eu were not included in the formulas because they have the potentials to exhibit an oxidation state. The geometric method was used to calculate Ce and Eu anomalies; this was achieved by assuming that the closest neighboring elements act linearly on log-linear plots [11,53]. Anomalies were calculated using the formulas:

(5) $$\delta Ce=\frac{2Ce_{n}}{La_{n}+Pr_{n}}$$

(6) $$\delta Eu=\frac{2Eu_{n}}{Sm_{n}+Gd_{n}}$$

where *δCe* and *δEu* are the measures of the anomalies for Ce and Eu, *n* implies PAAS-normalized concentrations.

## 5. Conclusions

This study provides the first quantitative investigation of a complete set of REE in sediment and mangrove *A. marina* in the central Red Sea mangrove ecosystem. This study reveals significant variation in REEs concentrations across the six mangrove ecosystems in the central Red Sea investigated, with high concentrations in Al lith (LT) and South Jeddah (SJ) mangrove ecosystems. This may be attributed to variations in anthropogenic sources at the catchment or in the mangrove ecosystems. Based on the relationship between REE values and sediment grain sizes, clay silt and coarse grain particles had a high contribution to the total variation; however, clay silt sediment grain size shows obvious strong positive relationships with REE concentrations. Although BCF shows hypo-accumulation of REE by *A. marina*, the pattern of REE distribution in leaves across the mangrove ecosystems was similar to that of sediment. The geo-accumulation index revealed strong contamination (4 ≤ I_geo_ ≥ 5) of sediment with La, Ce, Pr, Sm, Gd, Ho, Er, Yb, and Lu and moderate contamination (1 ≤ I_geo_ ≤ 3) with Tm, Tb, and Dy.

REE fractionating caused remarkable enrichment of lighter REEs (MREE and LREE) over the HREE in the mangrove ecosystems investigated. This was supported by positive and negative values of multi-elemental ratios R_(M/L)_ and R_(H/M)._ Even though concentrations of individual REE were higher at LT and SJ, higher values of (La/Yb)n (0.72) in Rabigh (RB), and (Sm/La)n (2.34) and (Yb/Sm)n (1.12) in Mastorah (MA) suggests the predominance of LREE at RB and MREE and HREE at MA. Positive Eu anomalies were recorded in the mangroves, which may be due to dominant reducing conditions in sediments. The results from this study are new evidence for REE accumulation in sediment and *A. marina* in mangroves of the central Red Sea. Therefore the REE values in this study can be used as background values for further investigation and designing of environmental monitoring studies and management policies by stakeholders of this important ecosystem.

## Figures and Tables

**Table 1:** Sediment grain sizes for six mangrove ecosystems in the central Red Sea.

Grain Size	Al Lith	South Jeddah	Dahaban	Thuwal	Rabigh	Mastorah
CS (0.063–2 mm)	83.53 ± 12.54	88.83 ± 17.48	65.37 ± 13.95	52.53 ± 9.06	45.50 ± 7.52	72.48 ± 11.66
CSTP (<0.063 mm)	15.97 ± 2.55	10.53 ± 1.32	34.42 ± 2.17	47.15 ± 6.82	54.29 ± 9.31	27.06 ± 5.16
G (>2 mm)	0.49 ± 0.05	0.63 ± 0.06	0.20 ± 0.08	0.31 ± 0.06	0.20 ± 0.01	0.45 ± 0.03
Texture	Sand	Sand	Sand	Sand	Loamy sand	Sand

CS—Coarse sandy, CSTP—Clay and Silt particles, G—Gravels.

**Table 2:** Rear earth elements concentrations (mg/kg) and fractions in central Red Sea.

	Al Lith	South Jeddah	Dahaban	Thuwal	Rabigh	Mastorah	Average	*p*-Value
La	16.54 ± 0.32	12.59 ± 0.1	2.59 ± 0.03	2.26 ± 0.01	3.01 ± 0.1	4.36 ± 0.10	6.89 ± 1.50	0.001
Ce	37.36 ± 0.45	27.74 ± 0.19	5.84 ± 0.15	5.45 ± 0.15	7.88 ± 0.20	9.85 ± 0.12	15.69 ± 3.51	0.000
Pr	4.73 ± 0.02	3.73 ± 0.10	0.77 ± 0.03	0.66 ± 0.03	0.98 ± 0.03	1.43 ± 0.01	2.05 ± 0.07	0.000
Nd	21.60 ± 0.20	16.12 ± 0.19	3.36 ± 0.06	2.84 ± 0.02	4.20 ± 0.02	6.35 ± 0.11	9.08 ± 1.21	0.003
Sm	4.65 ± 0.10	3.43 ± 0.07	0.74 ± 0.02	0.61 ± 0.03	0.84 ± 0.01	1.48 ± 0.01	1.96 ± 0.06	0.000
Eu	1.34 ± 0.06	0.97 ± 0.03	0.19 ± 0.01	0.17 ± 0.02	0.19 ± 0.01	0.43 ± 0.01	0.55 ± 0.02	0.000
Gd	4.45 ± 0.21	3.20 ± 0.14	0.70 ± 0.02	0.58 ± 0.03	0.77 ± 0.03	1.48 ± 0.01	1.86 ± 0.06	0.000
Tb	0.74 ± 0.03	0.50 ± 0.01	0.10 ± 0.01	0.09 ± 0.01	0.11 ± 0.01	0.23 ± 0.01	0.30 ± 0.01	0.002
Dy	4.09 ± 0.06	2.84 ± 0.01	0.63 ± 0.01	0.52 ± 0.03	0.68 ± 0.02	1.46 ± 0.01	1.70 ± 0.05	0.000
Ho	0.79 ± 0.02	0.57 ± 0.01	0.13 ± 0.01	0.10 ± 0.01	0.13 ± 0.01	0.29 ± 0.01	0.33 ± 0.02	0.000
Er	2.28 ± 0.02	1.58 ± 0.01	0.32 ± 0.01	0.25 ± 0.03	0.32 ± 0.01	0.81 ± 0.02	0.93 ± 0.04	0.000
Tm	0.37 ± 0.02	0.25 ± 0.02	0.06 ± 0.01	0.04 ± 0.01	0.05 ± 0.01	0.13 ± 0.01	0.15 ± 0.01	0.004
Yb	2.25 ± 0.02	1.63 ± 0.03	0.31 ± 0.01	0.26 ± 0.01	0.31 ± 0.01	0.84 ± 0.02	0.94 ± 0.03	0.000
Lu	0.36 ± 0.03	0.23 ± 0.02	0.05 ± 0.01	0.03 ± 0.01	0.04 ± 0.01	0.12 ± 0.02	0.14 ± 0.01	0.000
∑REE	101.53 ± 7.45	75.38 ± 5.55	15.79 ± 2.01	13.85 ± 1.65	19.50 ± 3.04	29.27 ± 3.92	42.56 ± 6.06	0.001
(La/Yb)n	0.54 ± 0.02	0.57 ± 0.03	0.61 ± 0.02	0.63 ± 0.04	0.72 ± 0.06	0.38 ± 0.01	0.57 ± 0.04	0.007
(Sm/La)n	1.94 ± 0.06	1.88 ± 0.04	1.96 ± 0.06	1.85 ± 0.05	1.91 ± 0.05	2.34 ± 0.14	1.98 ± 0.04	0.005
(Yb/Sm)n	0.95 ± 0.03	0.94 ± 0.08	0.84 ± 0.04	0.86 ± 0.03	0.73 ± 0.06	1.12 ± 0.09	0.91 ± 0.05	0.006
R_(M/L)_	0.24 ± 0.01	0.20 ± 0.01	0.21 ± 0.03	0.21 ± 0.02	0.16 ± 0.04	0.30 ± 0.04	0.22 ± 0.02	0.032
R_(H/M)_	−0.04 ± 0.02	−0.05 ± 0.01	−0.05 ± 0.01	−0.13 ± 0.02	−0.15 ± 0.01	−0.01 ± 0.001	−0.07 ± 0.002	0.004
δCe	0.97 ± 0.04	0.93 ± 0.03	0.95 ± 0.05	1.03 ± 0.33	1.05 ± 0.41	0.90 ± 0.06	0.97 ± 0.04	0.014
δEu	1.38 ± 0.43	1.37 ± 0.40	1.26 ± 0.24	1.36 ± 0.34	1.09 ± 0.20	1.37 ± 0.21	1.31 ± 0.25	0.021

∑REE is the sum of rare earth elements, (La/Yb)n is the relationship between light and heavy REE using a single elemental ratio, (Sm/La)n is the relationship between medium and light REE using a single elemental ratio, (Yb/Sm)n is the relationship between heavy and medium REE using single elemental ratio, R_(M/L)_ is the relationship between medium and light REE using multi-elemental ratio, R_(H/M)_ is the relationship between heavy and medium REE using multi-elemental ratio, δCe and δEu are Cerium (Ce) and Europium (Eu) anomalies.

**Table 3:** Rear earth elements concentrations (mg/kg) and fractions in mangrove *A. marina* at the central Red Sea.

	Al Lith	South Jeddah	Dahaban	Thuwal	Rabigh	Mastorah	Average	*p*-Value
La	0.68 ± 0.11	1.11 ± 0.03	0.50 ± 0.01	0.43 ± 0.04	0.57 ± 0.06	0.71 ± 0.05	0.67 ± 0.09	0.000
Ce	2.02 ± 0.15	1.46 ± 0.13	0.95 ± 0.18	0.82 ± 0.11	1.15 ± 0.08	1.42 ± 0.19	1.30 ± 0.02	0.000
Pr	0.24 ± 0.04	0.17 ± 0.03	0.12 ± 0.03	0.10 ± 0.02	0.13 ± 0.01	0.16 ± 0.02	0.15 ± 0.01	0.012
Nd	0.95 ± 0.09	0.67 ± 0.07	0.45 ± 0.04	0.39 ± 0.02	0.55 ± 0.08	0.61 ± 0.10	0.60 ± 0.03	0.000
Sm	0.22 ± 0.04	0.16 ± 0.02	0.10 ± 0.03	0.09 ± 0.01	0.15 ± 0.01	0.13 ± 0.01	0.14 ± 0.02	0.005
Eu	0.05 ± 0.002	0.05 ± 0.002	0.04 ± 0.01	0.03 ± 0.001	0.04 ± 0.002	0.04 ± 0.001	0.04 ± 0.003	0.411
Gd	0.21 ± 0.03	0.14 ± 0.03	0.11 ± 0.02	0.08 ± 0.01	0.12 ± 0.01	0.13 ± 0.03	0.13 ± 0.01	0.011
Tb	0.04 ± 0.003	0.04 ± 0.001	0.03 ± 0.001	0.02 ± 0.001	0.03 ± 0.002	0.03 ± 0.001	0.03 ± 0.003	0.390
Dy	0.18 ± 0.02	0.12 ± 0.01	0.09 ± 0.003	0.09 ± 0.001	0.10 ± 0.02	0.11 ± 0.01	0.12 ± 0.01	0.012
Ho	0.05 ± 0.003	0.04 ± 0.001	0.03 ± 0.001	0.03 ± 0.002	0.03 ± 0.001	0.03 ± 0.002	0.04 ± 0.003	0.254
Er	0.10 ± 0.02	0.08 ± 0.002	0.06 ± 0.003	0.05 ± 0.001	0.05 ± 0.001	0.07 ± 0.002	0.07 ± 0.004	0.064
Tm	0.03 ± 0.007	0.03 ± 0.005	0.02 ± 0.004	0.02 ± 0.005	0.02 ± 0.006	0.02 ± 0.005	0.02 ± 0.001	0.843
Yb	0.08 ± 0.01	0.07 ± 0.01	0.05 ± 0.007	0.05 ± 0.007	0.06 ± 0.009	0.06 ± 0.008	0.06 ± 0.002	0.178
Lu	0.03 ± 0.007	0.03 ± 0.004	0.02 ± 0.006	0.02 ± 0.005	0.02 ± 0.004	0.02 ± 0.007	0.02 ± 0.001	0.898
∑REE	3.56 ± 0.22	3.02 ± 0.06	3.69 ± 0.23	2.57 ± 0.17	2.26 ± 0.05	5.29 ± 0.92	3.40 ± 0.44	0.003

## Data Availability

The data presented in this study are available on request from the authors.

## Supplementary Materials

The following are available online at https://www.mdpi.com/article/10.3390/plants10061233/s1, Table S1: Analytical results achieved on certified reference materials for sediment and leaves, Table S2: Classification of Sediment quality (Geo—accumulation Index).
