# Supplementary material for: Fractionation and Distribution of Rare Earth Elements in Marine Sediment and Bioavailability in Avicennia marina in Central Red Sea Mangrove Ecosystems
Source: Plants (Basel). 2021 Jun 17;10(6):1233. doi: 10.3390/plants10061233 (PMC8235498; doi:10.3390/plants10061233)
Supplement: Supplementary file 1 [file plants-10-01233-s001.zip › plants-1247681-supplementary.pdf]

Supplementary Material

1 Supplementary Tables

Table S1: Analytical results achieved on certified reference materials for sediment and leaves.

Sediment	Elements (mg/kg)	GSS-1			Leaves	GSV-2		
		Certified	Experimental	Recovery (%)		Certified	Experimental	Recovery (%)
	**La**	34.00±4.47	33.20±4.21	97.65		1.25±0.02	1.26±0.11	100.80
	**Ce**	70.00±17.11	68.80±7.22	98.29		2.20±0.01	2.26±0.92	102.73
	**Pr**	7.50±0.25	7.45±0.52	99.33		0.29±0.04	0.30±0.03	103.45
	**Nd**	28.00±1.22	28.90±3.02	103.21		1.00±0.03	0.95±0.02	95.00
	**Sm**	5.20±0.33	5.13±0.81	98.65		0.19±0.01	0.21±0.04	110.53
	**Eu**	1.00±0.01	0.98±0.02	98.00		0.45±0.02	0.51±0.03	113.33
	**Gd**	4.60±0.94	4.61±0.04	100.22		0.17±0.02	0.18±0.03	105.88
	**Tb**	0.75±0.03	0.75±0.03	100.00		0.29±0.001	0.31±0.02	106.90
	**Dy**	4.60±0.08	4.33±0.21	94.13		0.14±0.02	0.16±0.04	114.29
	**Ho**	0.87±0.02	0.84±0.05	96.55		0.13±0.002	0.14±0.02	107.69
	**Er**	2.60±0.15	2.52±0.43	96.92		0.08±0.004	0.09±0.01	112.50
	**Tm**	0.42±0.02	0.43±0.02	102.38		0.10±0.002	0.11±0.01	110.00
	**Yb**	2.70±0.33	2.55±0.03	94.44		0.06±0.002	0.07±0.004	116.67
	Lu	0.41±0.02	0.40±0.01	97.56		0.11±0.001	0.12±0.001	109.09

Table S2 Classification of Sediment quality (Geo – accumulation Index)

I_{geo} value	I_{geo} Class	Sediment Quality
<0	0	Uncontaminated
0–1	1	Uncontaminated to moderately contaminated
1–2	2	Moderately contaminated
2–3	3	Moderately to strongly contaminated
3–4	4	Strongly contaminated
4–5	5	Strongly to extremely contaminated
> 5	6	Extremely contaminated
